# The bi-directional relationship between growth stunting and early childhood caries: a rapid review

**DOI:** 10.3389/fpubh.2023.1234893

**Published:** 2023-12-11

**Authors:** Refine Nissa Aulia, Ratna Indriyanti, Arlette Suzy Setiawan

**Affiliations:** ^1^Dental Education Program, Faculty of Dentistry, Universitas Padjadjaran, Bandung, Indonesia; ^2^Department of Pediatric Dentistry, Faculty of Dentistry, Universitas Padjadjaran, Bandung, Indonesia

**Keywords:** growth stunting, malnutrition, caries, children, bidirectional relationship

## Abstract

**Objective:**

This review aims to determine the two-way relationship between stunting and ECC in developing countries worldwide.

**Methods:**

The procedure in this study was carried out using the Preferred Reporting Items for Systematic Reviews and Meta-Analysis-Rapid Review protocol. Article searches were carried out using databases Cochrane and PubMed. In addition, searches were also carried out using backward and forward snowballing techniques to identify articles that were not detected through database searches.

**Results:**

Nine observational epidemiological articles were found in children aged six months to six years, published in 2015–2020. Five articles discussed the features of ECC in stunted children, and the other five discussed stunting in children with caries in their primary teeth.

**Conclusion:**

Several studies found associations between severe ECC and stunting, with ECC and stunting as both independent and dependent variables, suggesting a bi-directional relationship, also supported by literature on the physiological relationship between oral health and nutrition.

## 1 Introduction

Currently, malnutrition is a formidable global challenge, directly or indirectly linked to mortality and disability (1). Various forms of malnutrition, such as wasting, underweight, and stunting, afflict populations worldwide. Among these, stunting is most prevalent among children under five, especially in developing nations, where approximately 80% of cases occur (2, 3). In 2018, the global tally of stunted children under five reached a staggering 149 million, with most cases concentrated in Asia, accounting for 81.7 million instances (4).

In this global concern, it is essential to zoom in on Indonesia. According to the 2021 Indonesia Nutrition Status Study, there has been a notable decrease in the stunting rate, from 27.7% in 2019 to 24.4%, which is a promising trend despite the challenges posed by the COVID-19 pandemic (5). However, this figure, though improving, remains significantly high, necessitating continued efforts for reduction, particularly in alignment with the initial two targets of the Sustainable Development Goals (SDGs) aimed at eradicating poverty, hunger, and malnutrition. The Indonesian government has undertaken several commendable initiatives to combat extreme poverty, setting a target of 0 % by 2024. While many of these programs have yielded positive results in the context of malnutrition, few have addressed oral health concerns, which also bear significant importance in the fight against malnutrition (6, 7). In Indonesia, child oral health remains a neglected aspect of child health and malnutrition programs. Remarkably, comprehensive Lancet series reports in 2003, 2008, 2013, and 2021 did not include oral health and dental caries discussions. This underscores the urgent need for child health and malnutrition initiatives to incorporate oral health promotion and caries treatment into their strategies, ultimately enhancing child nutrition status.

Stunting, a distinct manifestation of malnutrition in children, is characterised by chronic and inadequate growth in height and length. Unlike wasting, which primarily affects weight and muscle mass, or underweight, which focuses on low body weight for age, stunting is related explicitly to linear growth retardation. This condition has several clinical symptoms, including reduced body mass, weakened muscles, inhibited intellectual development, impaired wound healing, increased susceptibility to infections, and compromised immune function (8–10).

Furthermore, the repercussions of stunting are not confined to physical health alone. They extend to oral health, with stunted children often presenting with dental caries on their primary teeth. This multifaceted impact underscores the importance of understanding and addressing stunting within the broader context of child malnutrition.

Early Childhood Caries (ECC), or primary tooth caries, is a condition characterized by one or more decayed teeth or tooth surfaces, tooth loss resulting from decay, or teeth restored in children under six (11). According to Global Burden of Disease 2017 data, it is estimated that over 530 million children worldwide are affected by caries in their primary teeth (12, 13). Research indicates that ECC is more prevalent among malnourished children than those with adequate nutrition. This is primarily because chronic malnutrition can impede proper tooth development, leading to reduced enamel thickness and a weaker tooth structure due to disrupted ameloblast activity during the amelogenesis process (6, 14–16).

In addition to malnutrition, the lack of evidence-based oral hygiene habits also plays a significant role in the development of ECC. Inadequate tooth brushing with fluoride toothpaste, infrequent dental check-ups, and suboptimal oral hygiene practices contribute to the accumulation of plaque and bacteria on teeth, which can lead to the onset and progression of caries in primary teeth. Therefore, promoting evidence-based oral hygiene habits from an early age, such as regular brushing with fluoride toothpaste, is crucial in preventing ECC and ensuring the overall oral health of young children (6, 14–16).

Not only does research suggest that stunting impacts the severity of caries in children, but several other studies have also shown that the relationship between stunting and ECC is bidirectional. This statement means that the severity of caries can increase the risk of children experiencing malnutrition (17). This rapid review aims to analyze this two-way relationship between stunting and early childhood caries to provide valuable information for relevant government agencies when formulating policies related to stunting and oral health.

## 2 Materials and methods

Rapid reviews, while not bound by the strict protocols of traditional systematic reviews like PICOS and PRISMA, should nonetheless uphold transparency and rigour. To achieve this, we have adopted a modified approach, incorporating elements from PRISMA-RR and adhering to the essence of the PICOS framework (18–20). By doing so, we aim to maintain the quality and clarity of our rapid review, despite its inherent constraints.

In defining our review objectives more clearly, our primary aim is to inform and contribute to health policy development in Indonesia. To address this, we have laid out specific inclusion criteria that reflect the uniqueness of our research focus. Our Population (P) encompasses children aged 0–5 years in early childhood. The Intervention (I) examines the relationship between two conditions, where one condition serves as an “exposure.” In this context, exposure pertains to growth stunting, akin to an “intervention” in traditional terms. The Comparison (C) is drawn from children without growth stunting or, conversely, those without early childhood caries. Our Outcome (O) is centred on the development of early childhood caries or growth stunting. Finally, we have specified that our Study design (S) will involve observational studies, including cohort, case–control, and cross-sectional studies.

Furthermore, to provide a more comprehensive rationale for our choice of rapid review methodology, we have emphasised that our objective is to facilitate specific policy recommendations for Indonesia. This thematic approach serves as a unifying thread throughout our research. In light of this, we will ensure that this theme is prominently featured in the discussion section, reinforcing the connection between our rapid review and its policy implications for Indonesia.

### 2.1 Study selection

This study’s inclusion criteria were articles with an analytical epidemiological study design, including all type of observational studies, that discussed the relationship between stunting and ECC in developing countries. These articles had to be published in English between 2012–2020 and use the def-t index to assess caries in children’s teeth. Descriptive studies, review articles and grey literature were excluded from this review. Grey literature refers to published and unpublished materials and is typically found in higher education institutions/libraries such as university libraries, institutes, high schools, academies, and polytechnics.

### 2.2 Search strategy

Search using the PRISMA-RR protocol (Preferred Reporting Items for Systematic Reviews and Meta-Analysis-Rapid Review) (18, 21), an article search strategy through databases such as Scopus and PubMed. The keywords used are “Malnutrition,” “Undernutrition,” “Nutritional Status,” “Stunting,” “Early Childhood Caries,” “Caries,” “Tooth Decay,” “Children,” “Child,” and “Preschool Children” which are then combined using the boolean operators “AND” and also “OR.” In addition, a list of articles that cite selected articles (forward snowballing) and a list of references (backwards snowballing) (22) also checked for articles that needed to be detected when searching through the database using the boolean operator.

### 2.3 Data extraction

After obtaining articles that match the inclusion criteria, data such as title, author, year of publication, research objectives, country, and research methodologies such as study design, sample, variable measurement indicators, research results and research conclusions are extracted and presented in tabular form.

### 2.4 Quality assessment

The quality of the selected articles was assessed by two reviewers using the NIH Quality Assessment Tools (23), of which the assessments were in the form of a questionnaire. Each assessment question is answered using the code ‘Y’ for yes, ‘N’ for no, ‘CD’ for cannot determine, ‘NR’ for not reporting, or ‘NA’ for not applicable. For the final assessment, each article is assessed using a rating category such as “good,” “average,” or “poor.” The differences of opinion between reviewers will be discussed further until a mutual agreement is reached.

### 2.5 Data synthesis

The quantitative narrative review approach is applied as a data synthesis strategy. A more intuitive systematic review refers to a summary and then includes a synthesis of the results of various studies with quantitative data using words (24). In this review, the presentation of data is grouped into two: articles with stunting as the independent variable and articles with ECC as the independent variable.

## 3 Result

### 3.1 Search result through the database

**Figure 1:**
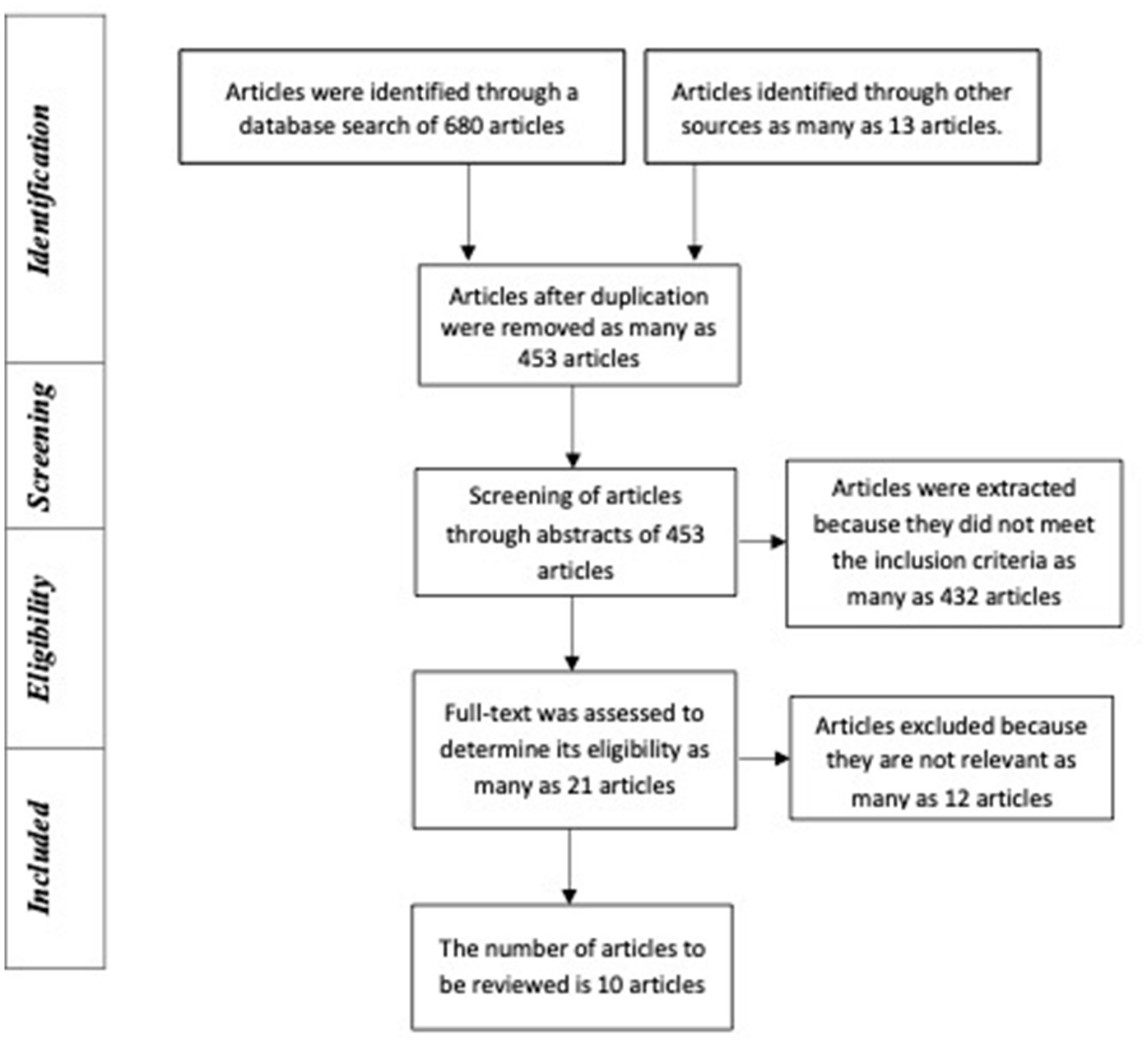
PRISMA flowchart.

After an initial search through two databases, 286 articles were found in PubMed, and another 394 were from Scopus. These articles were then removed by duplication so that there were 442 articles remaining. Furthermore, screening was carried out based on the title and 140 articles, which were then examined by abstracts. One hundred twenty-two articles had to be extracted because they did not meet the inclusion criteria, leaving 21 eligible for the overall review process. Of the 21 articles that have been reviewed as a whole, 12 articles did not meet the requirements, leaving nine articles for further review (Figure 1).

### 3.2 Search with the snowballing technique

Of the eight articles obtained through a database search, each was thoroughly examined, including the list of references and the articles cited. Following the search, we identified an additional 13 articles. Subsequently, we eliminated duplicate entries among these 13 articles, resulting in 11 unique articles. Of these 11 articles, only three met the criteria for a full-text review, and just one additional article met the inclusion criteria for further consideration in our research process.

### 3.3 Study characteristics

#### 3.3.1 Country and study design

A summary of the research findings can be seen in Table 1. Based on the country where the research took place, the continent of Asia was the area that produced the most articles (25, 29–33), followed by the continent of South America (26, 28), and the African continent (27, 34). Out of ten articles, only three did not use cross-sectional as the study design but used longitudinal and quasi-experimental studies (28, 31). The longitudinal and quasi-experimental studies reported a two-way relationship between stunting and ECC.

**Table 1:** Result.

No	Source			Purpose	Methodology			Result	Conclusion	Article quality
	Title	Author and reference	Country		Study design	Sample	stunting and ECC assessment			
	Stunting as an independent variable									
1.	Analysis of dental caries & gingivitis with the occurrence of stunting in children in Makassar City (Tamalanrea subdistrict)	Achmad et al. (25)	Indonesia	Knowing the incidence of caries and gingivitis with stunting in children living in the Tamalanrea area, Makassar.	Cross-sectional	208 children aged 2-5 years	Body height/age; def-t	Def-t index in stunted children is higher than in normal children (Mean: 7.37, SD: 4.56, *p*-value = 0.001)	There is a relationship between stunting and the level of child caries at the Tamalanrea Health Center, Makassar.	Poor
2.	Chronic malnutrition and oral health status in children aged 1 to 5 years: an observational study.	Vieira et al. (26)	Brazil	Assess the effect of chronic malnutrition on the oral health status of children aged 1-5 years	Cross-sectional	82 children aged 12-71 months	*Z*-scores; dmft index	No statistically significant relationship was found between nutritional status and the DMFT index (*p* > 0.05; *r* = −0.091)	No significant differences in ECC were found between the different nutritional status categories.	Good
3.	Association between early childhood caries and malnutrition in a sub-urban population in Nigeria.	Folayan et al. (27)	Nigeria	Knowing whether there is a relationship between ECC and malnutrition in children under 6 years old in Ile-Ife, Nigeria	Cross-sectional	370 children under 6 years old	*Z*-scores; dmft index	In model three, when confounding variables were controlled for (gender, oral hygiene status, frequency of sugar consumption, mother’s knowledge of caries prevention), it was found that there was a relationship between nutritional status and ECC (*p* = 0.02; APR: 0.14; 95 % CI: 0.03–0.69)	There is a significant association between the occurrence of ECC in children under 6 years of age who are stunted.	Good
4.	Early childhood caries and malnutrition: Baseline and two-year follow-up results of a community-based prevention intervention in rural Ecuador	Sokal-Gutierrez et al. (28)	Ecuador	Assessing the cross-sectional and longitudinal relationships between ECC and malnutrition in the context of community-based interventions designed to prevent ECC and malnutrition from birth.	Quasi experimental with a follow-up time of 2 years.	1,575 children aged 6 months-6 years	*Z*-scores; dmft index, caries depth	• A reduction in the incidence of caries was found in children who participated in the intervention during the child’s infancy, starting from 1-3 years of age at follow-up. • A 1 year old child’s caries experience is halved. Prevalence decreased from 53.8% to 30.3%, and dmft from 2.1 to 0.9. • The caries experience of children aged 2 years is reduced by one third in the sample. Prevalence decreased from 82% to 60%, dmft from 5.3 to 3, and deep caries from 19% to 12%.	After two years of follow-up to the community-scale preventive intervention, there was a significant reduction in the experience of ECC as well as malnutrition.	Good
5.	Stunting malnutrition associated with severe tooth decay in Cambodian Toddlers	Renggli et al. (29)	Cambodia	Examines the relationship between severe dental caries and anthropometric changes over a one-year period, in children under 2 years of age at baseline	Secondary analysis of a longitudinal cohort study	1,307 children aged <24 months	*Z*-sxores, dmft	There were 1,595 children who met the inclusion criteria and 1,307 (81.9%) were followed after one year. At baseline, 14.4% of the children had severe dental caries, 25.6% presented with stunted growth. 17.6% of the children transitioned from healthy status to a low height-for-age over the observation period. Children with severe dental caries had nearly double the risk (OR = 1.8; CI 1.0–3.0) of making that transition	Severe caries experience was associated with poorer childhood growth and, as such, could be an underinvestigated contributor to stunting.	Good
	ECC as independent variable									
6.	Associations between child snack and beverage consumption, severe dental caries, and malnutrition in Nepal	Zahid et al. (30)	Nepal	To investigate the relationship between food consumption habits of children in Nepal and caries, and between the severity of caries and nutritional status	Cross-sectional	273 children aged 6 months to 12 years	*Z*-scores; index dmft, *SiC*, pufa+PUFA	In younger children (age 6 months – 6 years), no association was found between S-ECC and the risk of malnutrition (Mean: −1.20; Mean difference: 0.13; 95% CI: 0.64, 0.38; *p* = 0.609)	There is no relationship between severe dental caries and the risk of malnutrition.	Good
7.	The bidirectional relationship between weight, height and dental caries among preschool children in China	Shen et al. (31)	China	Knowing the relationship between baseline caries and changes to body height and weight	Longitudinal Study with an average follow-up time of 9.73 months	1,111 pre-school age children	*Z*-scores; dmft index	Caries (baseline) was negatively and significantly related to changes in HAZ score in the model that had not been adjusted for confounding variables (coefficient: −0.03; 95% CI: −0.04,0.01). Likewise for adjusted factors (coefficient: −0.02; 95% CI: −0.04,0.01)	There is a negative relationship between the baseline dmft index and significant changes in HFA.	Good
8.	Early childhood oral health and nutrition in urban and rural Nepal.	Tsang et al. (32)	Nepal	Assessing social and behavioral risk factors for the incidence of ECC in rural & urban populations, and the relationship between caries and child nutrition in Nepal.	Cross-sectional	836 children aged 6 months to 6 years	*Z*-scores; dmft index and caries depth	The severity of child caries as measured by the dmft index correlated with the occurrence of mouth pain and both chronic and acute malnutrition (HAZ score) (*p* < 0.0001)	Severe caries has a relationship with the occurrence of malnutrition, especially in children who live in rural areas.	Average
9.	Early childhood caries, mouth pain, and nutritional threats in Vietnam	Khanh et al. (33)	Vietnam	Investigate the relationship between ECC, mouth pain and nutritional status in children aged 1-6 years in south and central Vietnam.	Cross-sectional	593 children aged 1-6 years	Z-scores, dmft and PUFA indices.	There was a high prevalence of dental caries (74.4%), mostly untreated, and mouth pain (47.1%). Moderate correlations were found between parents’ and children’s consumption of soda (*p* = 0.361; *p* < 0.001) and salty snacks (*p* = 0.292; *p* < 0.001). Severity of ECC was associated with decreased weight-and body mass index-for-age *z*-scores. Presence of pulp-involved caries was associated with strikingly lower height-for-age (mean difference = 0.66; *p* = 0.001), weight-for-age (mean difference = 1.17; *p* < 0.001), and body mass index-for-age (mean difference = 1.18; *p* < 0.001) *z*-scores. Mouth pain was associated with lower body mass index-for-age *z*-scores (mean difference = 0.29; *p* = 0.013).	ECC might negatively affect children’s nutritional status, which might be mediated by the depth of decay, chronic inflammation, and mouth pain. Family-based and prevention-oriented nutrition and oral health programs are needed and should start during pregnancy and infancy	Average

#### 3.3.2 Relationship of ECC in stunted children

Five articles discussed the relationship between ECC (the dependent variable) in children with stunting (the independent variable), with two statistical reporting estimates of the association between the two variables (27, 31). It is also known that four articles are cross-sectional studies, and one is longitudinal. In addition, the four articles show varying numbers for the number of samples, ranging from 82 to 1,575 children (25–28).

#### 3.3.3 Stunting relationship with ECC

Three cross-sectional studies (30, 32, 33) and two longitudinal study (29, 31) investigated the description of stunting cases (the dependent variable) as measured using HAZ-scores in children with ECC cases (the independent variable), with a sample range from 273 to 1,307 children. Of the five articles, only one article reported statistical estimates regarding the relationship between stunting and ECC (31).

#### 3.3.4 Quality of studies

From the assessment results of the two reviewers, it was found that six articles had good ratings (26–31), two articles had average ratings (32, 33), and one other article had a poor rating because of statistical methods, data collection methods, and confounding variables were not taken into account (Table 1) (25).

## 4 Discussion

This study is the first rapid review exploring the bidirectional relationship between stunting and Early Childhood Caries (ECC) in developing countries. The findings of our review reveal a compelling connection between stunting and ECC, with four articles demonstrating a significant relationship between these two variables (25, 27–29, 31). Malnutrition among children can reduce the intake of essential micronutrients, such as vitamins A and D, calcium, phosphorus, iron, and other crucial proteins. Both macro-and micronutrient deficiencies can impact the function of salivary glands, resulting in changes in saliva composition. These alterations can disrupt the remineralisation process and perpetuate the demineralisation process, ultimately leading to dental caries in children’s teeth (25, 27, 31). Additionally, socioeconomic factors and parental knowledge about nutrition and oral health also play a role in caries development among stunted children (25, 35, 36).

Study on the analysis that carried out by comparing specific categories of malnutrition (stunting, wasting, underweight, obesity) with children who did not experience any of these categories by Folayan et al. (27) resulted that there was no bidirectional relationship between stunting and ECC. Another study reports the results of Vieira’s research, which also states that stunting is not related to the occurrence of caries in children but is influenced by other confounding factors, which are risk factors for the two variables, both stunting and ECC (26).

In a cross-sectional study by Tsang et al. of children aged 6 months to 6 years in Nepal, the severity of caries assessed using the DMFT index has a relationship with the tendency of children to experience mouth pain and malnutrition, both chronic (stunting) and acute (wasting) (32). A quasi-experimental study of prevention intervention for early childhood caries in Ecuador by Sokal-Guiterrez et al. demonstrated reduction in caries and stunting, particularly in children who began the intervention in the first two years of life (28). This happens because several cases of malnutrition in this population are related to the target of the intervention factors, namely the frequency of consumption of junk food, S-ECC, which involves acute and chronic infection or inflammation, and also sore mouth. Of the three groups that had been given the intervention, the children with moderate caries development benefited the most from this intervention because children can experience caries-related malnutrition. Whereas in the children with low caries development, the prevalence of malnutrition was high from the start, and the average HAZ-score did not improve because children in this group may suffer from malnutrition unrelated to caries. An association between malnutrition and caries can occur even though the association is not statistically significant. S-ECC in children can affect the child’s ability to sleep, interfere with sleep quality, cause loss of appetite, and accelerate the adverse effects of other risk factors on nutritional status through infection or chronic inflammation (31).

In a cross-sectional study by Zahid et al. of children aged 6 months to 12 years in Nepal, S-ECC shows no statistically significant association with the risk of malnutrition in the group of children under six years as measured by the dmft index, but a relationship is found in the group of children aged 6–12 years whose caries is measured using the *SiC* index (Significant Caries Index) and in the entire population (age six months to less than 12 years) as measured by the pufa+PUFA index (30). The average HAZ score did not have a significant difference based on the severity of dental caries measured using the dmft index. The relationship between low HAZ-score was only found in children with caries depth that reached the pulp compared to children who did not have caries. Their research also found that caries severity was unrelated to stunting; caries had a significant relationship with wasting and being underweight. According to the authors, stunting is related to maternal malnutrition and malnutrition in children under two years of age. Meanwhile, wasting is associated with malnutrition at all ages, including in children over two years old (33).

In Renggli et al. 1-year longitudinal follow-up study of children under age 2 at baseline in Cambodia, 14.4% had severe dental caries, and 25.6% presented with stunted growth. After a year follow-up, 17.6% of the children transitioned from healthy to low height-for-age over the observation period. Children with severe dental caries had nearly double the risk (OR = 1.8; CI 1.0–3.0) of making that transition. Severe caries experience was associated with poorer childhood growth and, as such, could be an underinvestigated contributor to stunting. The study highlights the need to prevent and treat early childhood tooth decay as a vital part of programs to prevent child undernutrition and NCDs and promote optimal growth and development during a critical stage of life (29).

## 5 Strength and limitation

The same goes for this rapid review, which has some limitations. First, almost the entire review process is performed by a single reviewer, which increases the risk of bias. Literature may be ignored when searching for articles using only two databases. Furthermore, introducing language restrictions limited to English also risks overlooking essential lessons to be learned from articles published in other languages. Despite its limitations, the review has the strength of meeting high methodological standards as it assessed the quality and risk of bias of the studies. In addition, this review provides essential information on the relationship between developmental delay and ECC, which can serve as input for dentists and health system policymakers in developing strategies to improve children’s dental and oral health and nutritional status. Implications for future research: The study is hoped to be conducted with more detailed and precise methods and longitudinal study designs involving large populations.

## 6 Conclusion

The literature review reveals substantial evidence supporting a bidirectional relationship between severe Early Childhood Caries (ECC) and stunting in children. This complex interplay is evident in both longitudinal and cross-sectional studies, although it’s important to note that most of the included studies are cross-sectional, limiting the establishment of definitive causal relationships. The observed bidirectional relationship aligns with existing research on the physiological mechanisms connecting poor oral and nutritional health.

In summary, this review concludes that there is a compelling basis to consider a bidirectional relationship between severe ECC and stunting in children. It underscores the need for further research employing rigorous study designs to investigate causation more definitively. Additionally, interventions addressing both oral and nutritional health are warranted to mitigate the potential adverse effects of this relationship on child development and well-being.

## Data availability statement

The raw data supporting the conclusions of this article will be made available by the authors, without undue reservation.

## Author contributions

AS conceived the research idea, designed the study, and was responsible for coordinating the data collection process. She played a key role in revising and editing the manuscript for intellectual content. RI contributed to the refinement of the study design, performed statistical analyses on the collected data, and assisted in interpreting the results. RA conducted an extensive literature review to provide a theoretical background for the study. She also contributed to data analysis, created data visualisations, and helped to shape the paper’s discussion section. She conducted mindfulness meditation sessions with participants and wrote the initial draft of the manuscript. All authors contributed to the article and approved the submitted version.

## References

[1] World Health Organization. Essential nutrition actions: Improving maternal, newborn, infant and young child health and nutrition [internet]. Vol. 66, World Health Organizationr. (2013). Available at: https://www.who.int/publications/i/item/9789241505550 25473713

[2] Suryanti N Setiawan AS. Developing an instrument to measure maternal knowledge and attitude of Oral health on children under 3 years. Eur J Dent. (2021) 15:624–9. doi: 10.1055/s-0041-1725579 34041729 PMC8630960

[3] Tiwari R Ausman LM Agho KE. Determinant of Stunting and Severe Stunting Among Under-Fives: Evidence from the 2011 Nepal Demographic and Health Survey. BMC Pediatr. (2014) 78:464–9. doi: 10.1186/1471-2431-14-239 PMC4263111 25262003

[4] UNICEF – WHO – World Bank Group joint child malnutrition estimates. Levels and trends in child malnutrition: Key findings of the 2021 edition of the joint child malnutrition estimates. [internet]. (2021). Available at: https://www.who.int/publications/i/item/9789240025257

[5] Kementerian Kesehatan Republik Indonesia. Buku Saku Hasil Studi Status Gizi Indonesia (SSGI) Tahun 2021. Jakarta: Kementerian Kesehatan Republik Indonesia (2021). 133 p.

[6] Setiawan AS Indriyanti R Suryanti N Rahayuwati L Juniarti N. Neonatal stunting and early childhood caries: a mini-review. Front Pediatr. (2022):1–8. doi: 10.3389/fped.2022.871862/full PMC9339654 35923789

[7] Mayfitriana Z Suwargiani AA Setiawan AS. Growth stunting prevention in Indonesia: dentist knowledge and perception. Eur J Dent. (2022) 17:642–8. doi: 10.1055/s-0042-1757465 36351456 PMC10569837

[8] Sheetal A Hiremath VK Patil AG Sajjansetty S Sheetal KR. Malnutrition and its oral outcome – a review. J Clin Diagn Res. (2013) 7:178–80. doi: 10.7860/JCDR/2012/5104.2702, PMID: 23449967 PMC3576783

[9] Setiawan AS Abhista N Andisetyanto P Indriyanti R Suryanti N. Growth stunting implication in children: a review on primary tooth eruption. European J Gen Dent. (2022) 11:007–16. doi: 10.1055/s-0042-1742357

[10] Sadida ZJ Indriyanti R Setiawan AS. Does growth stunting correlate with Oral health in children?: a systematic review. Eur J Dent. (2022) 16:32–40. doi: 10.1055/s-0041-1731887, PMID: 34598296 PMC8890921

[11] American Academy of Pediatric Dentistry. Policy on early childhood caries (ECC): classifications, consequences, and preventive strategies. The Reference Manual of Pediatric Dentistry Chicago, [Internet]. (2021);III:81–84. Available at: https://www.aapd.org/globalassets/media/policies_guidelines/p_eccconsequences.pdf

[12] James SL Abate D Abate KH Abay SM Abbafati C Abbasi N . Global, regional, and national incidence, prevalence, and years lived with disability for 354 diseases and injuries for 195 countries and territories, 1990-2017: a systematic analysis for the global burden of disease study 2017. Lancet. (2018) 392:1789–858. doi: 10.1016/S0140-6736(18)32279-7, PMID: 30496104 PMC6227754

[13] Seow WK. Early childhood caries. Pediatr Clin N Am. (2018) 65:941–54. doi: 10.1016/j.pcl.2018.05.004, PMID: 30213355

[14] Dimaisip-Nabuab J Duijster D Benzian H Heinrich-Weltzien R Homsavath A Monse B . Nutritional status, dental caries and tooth eruption in children: a longitudinal study in Cambodia, Indonesia and Lao PDR. BMC Pediatr. (2018) 18:11. doi: 10.1186/s12887-018-1277-6 30217185 PMC6137874

[15] Yohana S Indriyanti R Suryanti N Rahayuwati L Juniarti N Setiawan AS. Caries experience among children with history of neonatal stunting. Eur J Dent. (2022) 17:687–92. doi: 10.1055/s-0042-1750775 36075267 PMC10569887

[16] Almoudi MM Hussein AS Abu Hassan MI Schroth RJ. Dental caries and vitamin D status in children in Asia. Pediatr Int. (2019) 61:327–38. doi: 10.1111/ped.13801 30740822

[17] Athavale P Khadka N Roy S Mukherjee P Mohan DC Turton B . Early childhood junk food consumption, severe dental caries, and undernutrition: a mixed-methods study from Mumbai, India. Int J Environ Res Public Health. (2020) 17:1–17. doi: 10.3390/ijerph17228629 PMC7699964 33233797

[18] King VJ Stevens A Nussbaumer-Streit B Kamel C Garritty C. Paper 2: performing rapid reviews. Syst Rev. (2022) 11:151. doi: 10.1186/s13643-022-02011-5 35906677 PMC9338520

[19] Tricco AC Langlois E V. Straus SE. Rapid reviews to strengthen health policy and systems: A practical guide [internet]. Geneva; (2017). Report No.: 978 92 4 151276 3. Available at: https://ahpsr.who.int/publications/i/item/2017-08-10-rapid-reviews-to-strengthen-health-policy-and-systems-a-practical-guide

[20] Aronson JK Heneghan C Mahtani KR Plüddemann A. A word about evidence: “rapid reviews” or “restricted reviews”? BMJ Evid Based Med. (2018) 23:204–5. doi: 10.1136/bmjebm-2018-111025, PMID: 29959158

[21] Moher D Liberati A Tetzlaff J Altman DG. Preferred reporting items for systematic reviews and Meta-analyses: the PRISMA statement. PLoS Med. (2009) 6:1–6. doi: 10.1371/journal.pmed.1000097 PMC3090117 21603045

[22] Wohlin C. Guidelines for snowballing in systematic literature studies and a replication in software engineering. Proceedings of the 18th International Conference on Evaluation and Assessment in Software Engineering. (2014) 1–10.

[23] National Heart Lung and blood institute. Study Quality Assessment Tools [Internet] (2021). Available at: https://www.nhlbi.nih.gov/health-topics/study-quality-assessment-tools

[24] Efron SE Ravid R. Writing the literature review: a practical guide (2019). 1st Edn. The Guilford Press, 32 p.

[25] Achmad H Handayani H Singgih MF Horax S Ramadhany S Setiawati F . Analysis of dental caries & gingivitis with the occurrence of stunting in children in Makassar city (Tamalanrea subdistrict). Syst Rev Pharm. (2020) 11:371–6.

[26] Vieira KA Rosa-Júnior LS Souza MAV Santos NB Florêncio TMMT Bussadori SK. Chronic malnutrition and oral health status in children aged 1 to 5 years: an observational study. Medicine (Baltimore). (2020) 99:1–7. doi: 10.1097/MD.0000000000019595 PMC7440136 32358344

[27] Folayan MO Arije O el Tantawi M Kolawole KA Obiyan M Arowolo O . Association between early childhood caries and malnutrition in a sub-urban population in Nigeria. BMC Pediatr. (2019) 19:433. doi: 10.1186/s12887-019-1810-2 31722683 PMC6852898

[28] Sokal-Gutierrez K Turton B Husby H Paz CL. Early childhood caries and malnutrition: baseline and two-year follow-up results of a community-based prevention intervention in rural Ecuador. BMC Nutr. (2016) 2:1–11.

[29] Renggli EP Turton B Sokal-Gutierrez K Hondru G Chher T Hak S . Stunting malnutrition associated with severe tooth decay in Cambodian toddlers. Nutrients. (2021) 13:290. doi: 10.3390/nu13020290, PMID: 33498508 PMC7909538

[30] Zahid N Khadka N Ganguly M Varimezova T Turton B Spero L . Associations between child snack and beverage consumption, severe dental caries, and malnutrition in Nepal. Int J Environ Res Public Health. (2020) 17:1–13. doi: 10.3390/ijerph17217911 PMC7672540 33126647

[31] Shen A Bernabé E Sabbah W. The bidirectional relationship between weight, height and dental caries among preschool children in China. PLoS One. (2019) 14:e0216227. doi: 10.1186/s40795-016-0110-6, PMID: 31039199 PMC6490928

[32] Tsang C Sokal-Gutierrez K Patel P Lewis B Huang D Ronsin K . Early childhood Oral health and nutrition in urban and rural Nepal. Int J Environ Res Public Health. (2019) 16:783–90. doi: 10.3390/ijerph16142456 31295932 PMC6678585

[33] Khanh LN Ivey SL Sokal-Gutierrez K Barkan H Ngo KM Hoang HT . Early childhood caries, mouth pain, and nutritional threats in Vietnam. Am J Public Health. (2015) 105:2510–7. doi: 10.2105/AJPH.2015.302798, PMID: 26469655 PMC4638248

[34] Folayan MO Oginni AB el Tantawi M Alade M Adeniyi AA Finlayson TL. Association between nutritional status and early childhood caries risk profile in a suburban Nigeria community. Int J Paediatr Dent. (2020) 30:798–804. doi: 10.1111/ipd.12645 32243034

[35] Indriyanti R Nainggolan TR Sundari AS Chemiawan E Gartika M Setiawan AS. Modelling the maternal Oral health knowledge, age group, social-economic status, and Oral health-related quality of life in stunting children. Int J Stat Med Res. (2021) 10:200–7. doi: 10.6000/1929-6029.2021.10.19

[36] Putri NA Setiawan AS Setiawan AS. Oral health attitude with the socioeconomic conditions of mothers with growth stunting children. Eur J Dent. (2022). doi: 10.1055/s-0042-1757213, PMID: 36307113 PMC10756825

